# Natural disasters and infectious disease in Europe: a literature review to identify cascading risk pathways

**DOI:** 10.1093/eurpub/ckz111

**Published:** 2020-06-06

**Authors:** Jonathan E Suk, Eleanor C Vaughan, Robert G Cook, Jan C Semenza

**Affiliations:** 1 European Centre for Disease Prevention and Control, Solna, Sweden; 2 Bazian, Economist Intelligence Unit, London, UK

## Abstract

**Background:**

Natural disasters are increasing in their frequency and complexity. Understanding how their cascading effects can lead to infectious disease outbreaks is important for developing cross-sectoral preparedness strategies. The review focussed on earthquakes and floods because of their importance in Europe and their potential to elucidate the pathways through which natural disasters can lead to infectious disease outbreaks.

**Methods:**

A systematic literature review complemented by a call for evidence was conducted to identify earthquake or flooding events in Europe associated with potential infectious disease events.

**Results:**

This review included 17 peer-reviewed papers that reported on suspected and confirmed infectious disease outbreaks following earthquakes (4 reports) or flooding (13 reports) in Europe. The majority of reports related to food- and water-borne disease. Eleven studies described the cascading effect of post-disaster outbreaks. The most reported driver of disease outbreaks was heavy rainfall, which led to cross-connections between water and other environmental systems, leading to the contamination of rivers, lakes, springs and water supplies. Exposure to contaminated surface water or floodwater following flooding, exposure to animal excreta and post-disaster living conditions were among other reported drivers of outbreaks.

**Conclusions:**

The cascade effects of natural disasters, such as earthquakes and floods, include outbreaks of infectious disease. The projection that climate change-related extreme weather events will increase in Europe in the coming century highlights the importance of strengthening preparedness planning and measures to mitigate and control outbreaks in post-disaster settings.

## Introduction

Natural disasters displace populations, damage infrastructure, hinder economic growth and activity, cause death and injury, and increase the risk of infectious disease outbreaks. Globally, in 2018, natural disasters affected 61.7 million people, caused 10 373 deaths and several billion US dollars in damages.^1^ The long-term trend in total mortality attributed to natural disasters appears to be decreasing,^1^ but there are myriad technical and political challenges in reporting verifiable data from such events.^2^ Moreover, the nature and effects of these disasters are becoming increasingly complex, due to factors such as climate change, population movement, economic interconnectivity and globalization.

These interdependencies contribute to the ‘cascade effect’ of natural disasters, which is emerging as a priority area for research and for cross-sectoral and cross-border preparedness.^3^^,^^4^ The cascade effect has been defined as ‘the dynamics present in disasters, in which the impact of a physical event … generates a sequence of events in human sub-systems that result in physical, social or economic disruption’.^5^ Examples of this effect globally include the 2019 outbreak of cholera following Cyclone Idai in Mozambique; the 2011 Fukushima triple disaster, which involved an earthquake, tsunami and radionuclear disaster and the 2010 Eyjafjallajökul volcanic eruptions, which led to an ash cloud that severely disrupted global air traffic.

In terms of the effects on health, natural disasters and their cascade effects can create serious public health challenges, e.g. if disaster relief operations and provision of health care are adversely affected by damage to critical infrastructure or disruption to supply chains.^6^ More specifically, natural disasters can result in disease outbreaks, because of the cascade effects on the diverse risk drivers of infectious diseases. These drivers include factors linked to globalization, climate change, intensive agriculture and changes in land use, and social and demographic changes.^7–9^ At the same time, infectious disease outbreaks can also have disruptive effects on society, including on trade, tourism, health care provision and even social cohesion, as the Ebola outbreak in West Africa demonstrated.^9^

Understanding how the cascade effects of natural disasters can lead to infectious disease outbreaks is critical to inform preparedness strategies, and this is reflected in global and European policy frameworks and policies. Health is a priority in the Sendai Framework for Disaster Risk and Reduction,^10^ which includes ‘Understanding disaster risk’ as a key objective, thus ensuring that disaster risk management is based on the best available evidence of disaster risks and their potential cascade effects. In Europe, assessment and management of disaster risks adheres to the principles of the Sendai Framework and includes policy instruments such as the EU Civil Protection Mechanism, the EU Directive on the assessment and management of flood risks and Decision 1082/2013/EU on serious cross-border threats to health, which specifically addresses the inter-sectoral dimension of preparedness and response planning (Article 4).

In this literature review, we assessed reported infectious disease outbreaks in Europe following two types of natural disasters, earthquakes and flooding, the drivers of these outbreaks, response measures implemented and lessons learned.

## Methods

### Scope of the review

The scope of the review was as follows.

Natural disasters: This review focussed on earthquakes and floods because of their importance in Europe and their potential to elucidate the pathways through which natural disasters can lead to infectious disease outbreaks. Earthquakes and flooding are two of six natural disasters that the European Commission has identified as posing the greatest threat to Europe; the others are extreme weather, forest fires, epizootics (animal or plant diseases) and pandemics.^3^ Between 2000 and 2017, 34 earthquakes occurred in 13 countries in Europe, mainly in Italy and Greece, affecting a quarter of a million people and causing 701 deaths and damage costing almost US$29 billion. Flooding is among the most frequent natural disasters in Europe and, between 1980 and 2013, nearly 1500 flood events were recorded, causing over 4700 deaths and damage costing EUR150 billion.^11^

Infectious disease categories: The review used ECDC’s organizational structure as framework for searching for categories of infectious diseases:^12^ anti-microbial resistance and health care-associated infections; emerging and vector-borne diseases; food- and water-borne diseases and zoonoses; influenza and other respiratory viruses; HIV, sexually transmitted infections (STI) and viral hepatitis; tuberculosis and vaccine-preventable diseases

Countries: The review included all countries in the European Union and European Economic Area as well as Albania, Bosnia and Herzegovina, the Former Yugoslav Republic of Macedonia, Montenegro, Serbia and Turkey, and Kosovo (this designation is without prejudice to positions on status, and is in line with UNSCR 1244 and the ICJ Opinion on the Kosovo Declaration of Independence).^13^^,^^14^

### Search strategy

For target countries, infectious diseases and selected disaster types, the search strategy focussed on the selected types of natural disasters, the selected infectious diseases and the relevant countries. The approach used to identify relevant literature was as follows:

We searched Embase.com, which includes the biomedical databases MEDLINE and EMBASE (see Supplementary table S1). Search terms were based on ECDC’s categories of infectious diseases (see above) and the WHO document ‘Communicable diseases following natural disasters: risk assessment and priority interventions’.^12^^,^^15^ To avoid excluding potentially relevant studies, we did not use a standard definition of what constituted an outbreak, relying instead on whether or not a study reported that an outbreak had occurred. For instance, some studies reported very small numbers of cases but did not explicitly refer to an ‘outbreak’. These reports may still be of public health importance. In addition, to ensure inclusion of as wide a range of studies as possible, we did not use search filters to identify specific study designs.

The design of the search strategy means that studies or reports of earthquakes or flooding events that did not discuss infectious disease (either because there were no outbreaks or because outbreaks were not reported) would not have been retrieved by these searches.

To complement this search strategy, we also invited selected ECDC experts to provide documents relevant to the review, which proved to be an effective way to identify ‘hard to find’ studies. In addition, we reviewed the list of references for an ECDC risk assessment paper that was included in the analysis^16^ and for three reviews identified in the call for evidence that did not meet our criteria for inclusion. These three reviews explored infectious diseases following flooding in Europe,^17^ the threat of infectious diseases after natural disasters in general^18^ and the risk of water-borne diseases after extreme events.^19^ The review of references yielded an additional three unique studies for inclusion.^20–22^

### Inclusion criteria and screening

Inclusion criteria: Studies were included that were published in English and published from 2005 onwards and that reported on infectious diseases (whether or not there was a reported outbreak) following an earthquake or related to a flooding event in the relevant countries.

**Figure 1:**
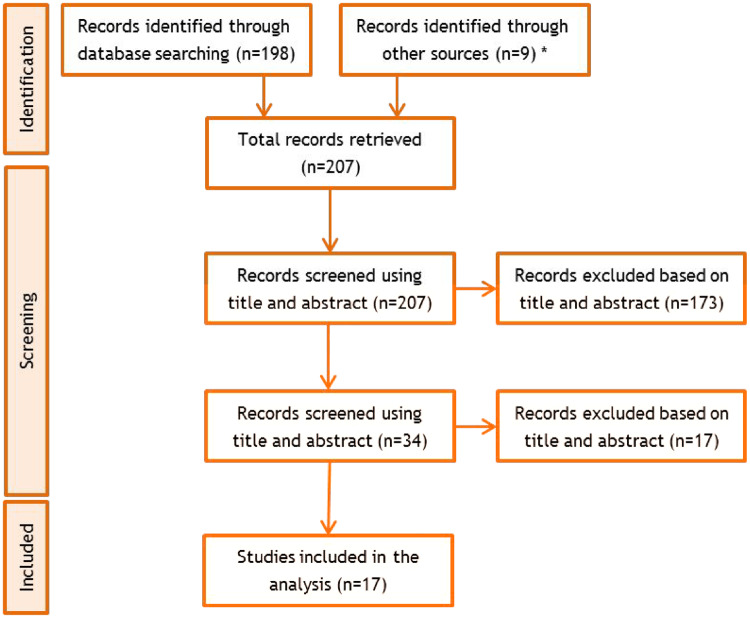
PRISMA diagram showing the flow of documents through the literature review

Screening: Studies identified were screened using a two-step process. The first step reviewed the title to exclude irrelevant studies (e.g. not related to earthquakes or flooding, not related to relevant countries). The second step reviewed the abstract to exclude any remaining irrelevant studies. In total, 198 records were retrieved, 34 full text articles were assessed for inclusion and 17 studies were included in the analysis (see figure 1).

## Results

Of the 17 included studies (see table 1), 4 reported on infectious disease events following earthquakes^23–26^ and the other 13 on infectious diseases related to flooding, usually as a result of heavy rainfall.^16^^,^^17^^,^^20–22^^,^^27–35^

**Table 1:** Included studies clustered by event type and pathogen/disease reported

Document title	Event	Disaster details reported (duration, numbers affected)	Post-event disease outbreak reported	Pathogen/disease covered in the report
Kaya et al.^23^	Earthquake	Two earthquakes (7.4 and 7.3 Richter scale) with an epicentre 10 km from Duzce. The earthquakes caused severe damage to the city, including property. People had to live in emergency accommodation (tents) or in the open, without guaranteed access to clean water or sanitation facilities	No	Hepatitis A & E
Nigro et al.^24^	Earthquake	6.3 magnitude earthquake (note: this is the same earthquake described by Petrazzi et al.^26^ below)	Yes	*Salmonella enterica*
Pérez-Martín et al.^25^	Earthquake	Two earthquakes in Spain, leading to the re-housing of 1424 people into emergency camps. A chickenpox outbreak was already in the community and had led to 163 reported cases in the 8 weeks preceding the earthquake	Yes	Chickenpox
Petrazzi et al.^26^	Earthquake	An earthquake measuring ∼6 on the Richter scale caused 308 deaths and 1600 people were injured. The only hospital in L'Aquila was damaged and patients/staff evacuated to a field hospital on the same day as the earthquake. Once complete, the field hospital had a 28-bed ward, radiology department, primary care facilities and a laboratory	Yes	Unclear—infectious diseases as a broad category
Christova and Tasseva^27^	Flooding	Heavy rainfall in 2014 led to flooding in several parts of Bulgaria	Yes	Leptospirosis
De Man et al.^28^	Flooding	Extreme rainfall on two occasions (>30 mm rainfall per hour for > 1h) causing surface flooding	No	Gastrointestinal, influenza-like illness, dermatological complaints
Desai et al.^20^	Flooding	Heavy rainfall led to waterlogged soil and strawberry pickers working in the rain	Yes	Leptospirosis
ECDC Rapid Risk Assessment^16^	Flooding	Flooding in Bosnia and Herzegovina, Croatia and Serbia	No	West Nile virus
Gertler et al.^29^	Flooding	Heavy rain led to river flooding in floodplains and the city centre of Halle (Saale), damaging sewer networks. Main sewer pipe has emergency spillways into the river at times of extremely high rainfall. The floodplains are in the centre of the city, where recreation activities take place such as swimming and picnicking in parks. These areas were cleaned and reopened mid-July onwards (at the start of summer holidays) during the summer season until end August	Yes	*Cryptosporidium hominis*
Harder-Lauridsen et al.^30^	Flooding	Heavy rainfall led to flooding and sewers overflowing. The following morning a triathlon event took place including a 3.8 km ocean swim	Yes	*Escherichia coli*, *Campylobacter jejuni*, *Giardia lamblia*
Hubálek et al.^21^	Flooding	Flooding in Prague and the rural areas along the rivers Vltava and Labe	No	West Nile virus, Sindbis virus, Batai virus and Tahyna virus among those tested for in the population
Radl et al.^22^	Flooding	Heavy rainfall preceded a triathlon including a swim in a man-made lake	Yes	Leptospirosis
Schmid et al.^31^	Flooding	Extreme rainfall led to flooding inside a hotel while a group of American tourists were checking in	Yes	Norovirus
Socolovschi et al.^32^	Flooding	The most severe period of rainfall in 10 years, leading to flooding over several days	Yes	Leptospirosis
Wasiński et al.^33^	Flooding	Two huge floods in the summer of 2010	No	Leptospirosis
Wójcik et al.^34^	Flooding	A torrential downpour lasting ca. 2.5 h at its most intense, leading to 135.4 mm of rain falling over 24 h, leading to flash flooding. 500 000 to >1 million people were affected, including insurance claims of ∼6.2 billion DKK (>Euro833 million)	Yes	Not reported
Zasada et al.^35^	Flooding	Extensive flooding affected Europe, Poland saw the biggest floods in 160 years	No	Anthrax

Reports were identified from the following countries: Austria;^22^^,^^31^ Bosnia and Herzegovina, Croatia and Serbia;^16^ Bulgaria;^27^ Czech Republic;^21^ Denmark;^30^^,^^34^ France;^32^ Germany;^20^^,^^29^ Italy;^24^^,^^26^ Poland;^33^^,^^35^ Spain;^25^ The Netherlands;^28^ Turkey.^23^

The findings from the analysis of the included studies are discussed below.

### Type and impact of infectious disease outbreaks after natural disasters

Of the 17 included studies, 10 related to food- and water-borne diseases and zoonoses, specifically, ‘leptospirosis’,^20^^,^^22^^,^^27^^,^^32^^,^^33^ anthrax,^35^*Cryptosporidium hominis*,^29^*Escherichia coli*, *Campylobacter jejuni* and *Giardia lamblia*,^30^ norovirus^31^ and *Salmonella enterica*.^24^ Two related to emerging and vector-borne diseases, specifically mosquito-borne diseases including West Nile virus, Sindbis virus, Batai virus and Tahyna virus.^16^^,^^21^ One related to HIV, STI and viral hepatitis, specifically hepatitis A and E,^23^ and one related to vaccine-preventable diseases, specifically, chickenpox.^25^ One report examined infectious diseases as a whole,^26^ one investigated gastrointestinal, influenza-like illness and dermatological complaints^28^ and one did not report on the pathogen or disease,^33^ so these could not be classified according to type. As the definition of outbreak was inconsistent across studies, an external standardized definition was not used in order to make the study as inclusive as possible.

Ten studies (see table 2) described outbreaks in post-disaster settings,^22^^,^^24–27^^,^^29–32^^,^^34^ and one study reported an outbreak following heavy rainfalls that led to flooded agricultural land.^20^ As noted above, this review defined whether an outbreak had occurred based on the authors’ reporting, not external classifications. Eight studies related to food- and water-borne diseases and zoonoses and one to vaccine-preventable diseases;^25^ two did not specify the pathogen or disease.^26^ No deaths were reported in any of the outbreaks.

**Table 2:** Included studies reporting outbreak impact—number of cases and fatalities

Document title	Event	Post-event disease outbreak	Reported number of cases	Reported deaths
Christova and Tasseva^27^	Flooding	Yes	Increase in the number of cases—from 12 in 2010, to 30 in 2014	None
Desai et al.^20^	Flooding	Yes	13 people hospitalized—11 with suspected leptospirosis. Mild symptoms reported in ∼20% of workers	None
Gertler et al.^29^	Flooding	Yes	24 cases, compared with a usual annual mean of 9	None
Harder-Lauridsen et al.^30^	Flooding	Yes	In 2010, 42% of triathlon participants reported diarrhoea and vomiting, only 8% in 2011	None
Nigro et al.^24^	Earthquake	Yes	155 children affected, 44 hospitalized	None
Pérez-Martín et al.^25^	Earthquake	Yes	9 cases—4 that led to the declaration of an outbreak, 5 after the vaccination programme had begun. 163 cases had been reported in the affected community in the 8 weeks preceding the earthquake	None
Petrazzi et al.^26^	Earthquake	Yes	Significant increase in hospital admissions for infectious diseases from 7.41% pre-earthquake to 27.18% post-earthquake, diagnosis rates in non-admitted patients also rose from 12.04 to 27.29%	None
Radl et al.^22^	Flooding	Yes	4 people admitted to hospital, one requiring haemodialysis due to signs of renal failure	None
Schmid et al.^31^	Flooding	Yes	26/36 exposed tourists fell ill, 10 presented to hospital. 6/10 firefighters also fell ill with vomiting and diarrhoea	None
Socolovschi et al.^32^	Flooding	Yes	3 confirmed cases	None
Wójcik et al.^34^	Flooding	Yes	22% of workers responding to the survey met the case definition of having an infectious disease. 16% visited a doctor and 7% missed a day or more of work as a result of illness	None

Two studies described cascading outbreaks following earthquakes. The earthquake in L’Aquila in Italy led to an outbreak of *Salmonella* among children.^24^ This is thought to have been due to the geological changes caused by the earthquake leading to contamination of the local spring, which was used to irrigate crops. The outbreak led to 155 children falling ill, with the highest incidence among those aged around 2 years, and 44 children being hospitalized. Another study looked at the incidence of infectious diseases in general following the L’Aquila earthquake.^26^ This found that admission rates for infectious diseases rose from 7.41% before the earthquake to 27.18% in the 2 months following the earthquake. Rates of people diagnosed with an infectious disease but not requiring hospitalization also rose, from 12.04 to 27.29%.

One study reported on an outbreak of chickenpox that had already been ongoing prior to an earthquake.^25^ In Spain, there had been 163 cases of chickenpox in Lorca in the 8 weeks preceding the earthquake that took place there. Following the earthquake, 1424 people were evacuated to a temporary emergency camp. Four cases of chickenpox were identified in the camp, leading to the declaration of an outbreak in the camp and the commencement of a vaccination programme; a further five cases were identified after the outbreak was declared.^25^

Eight studies described cascading risk pathways for outbreaks following flooding. Four of these reported on ‘leptospirosis’ outbreaks.^20^^,^^22^^,^^27^^,^^32^ In Bulgaria, unusually heavy rainfall in September 2014 led to an increase in the number of cases, from 12 in 2010 to 20 in 2014.^27^ In Germany, following heavy rainfalls, an outbreak of ‘leptospirosis’ among a group of strawberry pickers led to 13 being hospitalized and around 20% of the group overall reporting symptoms of ‘leptospirosis’.^20^ Following exposure to ‘leptospirosis’ while taking part in a triathlon in Austria, four people were admitted to hospital, including one who required haemodialysis due to signs of kidney failure.^22^ In France, there were three reported cases of ‘leptospirosis’ following heavy rain during a period where refuse collectors were on strike and uncollected rubbish in urban areas attracted rodents.^32^

Following heavy rainfall in Germany that led to the contamination of river water and recreational areas, there was an outbreak of 24 cases of *C. hominis*, compared with the usual annual mean of 9 cases.^29^ In Denmark, after seawater was inundated with sewage following heavy rainfall that overwhelmed the sewerage system, there was an outbreak of diarrhoea and vomiting among triathlon participants, caused by *E. coli*, *Ca. jejuni* and *G. lamblia*.^30^ In that year, 42% of triathlon participants reported diarrhoea and vomiting, whereas only 8% reported these symptoms after the following year’s competition. In the ‘leptospirosis’ outbreak in Austria, triathlon participants fell ill after swimming in a contaminated lake rather than seawater.^22^

Also in Austria, flooding in an hotel caused by heavy rainfall, including with water contaminated with sewage, resulted in 26 out of 36 hotel guests and 6 out of 10 firefighters who assisted in the clean-up falling ill with symptoms of norovirus.^31^ A study in Denmark looked at the occupational risk of workers helping in the clean-up following flooding in Copenhagen.^34^ It found that 22% of workers met the definition of having an infectious disease, 16% visited a doctor and 7% missed a day or more of work as a result. There was no difference in infection rates between workers who reported using personal protective equipment (PPE) and those who did not.

Some population groups may be more vulnerable or at risk of post-disaster infectious diseases than others. In Spain, young children in the emergency camp were particularly affected by chickenpox, mainly because the Spanish chickenpox immunization schedule applies to older children, which left these children unprotected.^25^ In Italy, where hygiene conditions were poor following the L’Aquila earthquake, older people were reported to be more vulnerable to infectious diseases, because of the effect of ageing on immunity.^26^ People working and living in agricultural settings may be particularly vulnerable to ‘leptospirosis’ following flooding or heavy rainfall. For example, in Bulgaria, men of working age were more likely to be infected with ‘leptospirosis’ than younger men or women, with cattle and pig farmers at the highest risk due to potential occupational exposure.^27^ In Germany, an outbreak of leptospirosis occurred among harvesters in a large strawberry field following heavy rainfall; harvesting with open hand wounds was one identified risk factor.^20^ Participants in triathlons and other water-based sports may also be more vulnerable to diseases such as leptospirosis due to prolonged exposure to potentially contaminated water;^36^ their vulnerability may also be increased because of the immunosuppressive effect of extreme sports.^22^

### Drivers of infectious disease outbreaks after natural disasters

Environmental factors were reported to be the main drivers of infectious disease outbreaks following natural disasters in Europe. The most reported driver was heavy rainfall, which led to cross-connections between water and other environmental systems, leading to the contamination of rivers, lakes, springs and water supplies. This contributed to five reported disease outbreaks following flooding.^22^^,^^27^^,^^29^^,^^30^^,^^32^ Exposure to contaminated surface water or flood water following flooding or heavy rainfall was the main driver of outbreaks in three outbreaks^20^^,^^31^^,^^34^ and exposure to sediment left behind after surface and flood water had subsided was also a driver in two outbreaks.^27^^,^^34^ One outbreak following an earthquake was attributed to geological changes that contaminated the local spring used to irrigate crops.^24^

Indirect contact with animal excreta and/or rodents was implicated in three of the four ‘leptospirosis’ outbreaks reported.^20^^,^^27^^,^^32^ This is not surprising as ‘leptospirosis’ is transmitted through exposure to animal urine. In one outbreak, exposure to ‘leptospirosis’ was via pest rodents,^32^ in another it was due to occupational contact with farm animals,^27^ and in another exposure to pest rodents and farm animals was implicated.^20^

Post-disaster living conditions were also implicated in two outbreaks following earthquakes.^25^^,^^26^ In Italy, this was due to extensive damage to residential properties and the resulting deterioration in living conditions immediately afterwards.^26^ In Spain, conditions in the emergency camp, including overcrowding, use of shared toilet and bathroom facilities and inadequate access to medical care had the potential to exacerbate an ongoing community outbreak of chickenpox.^25^

### Measures to prevent, mitigate and control outbreaks

Only 5 of the 17 included studies (4 of these 5 studies described an outbreak) described measures taken to prevent, mitigate and control outbreaks. It may be that prevention, mitigation and control measures were implemented in response to the other events reported on, as part of public health emergency preparedness plans, but it is not possible to determine this from the studies. The prevention, mitigation and control measures described or recommended in the five studies were generally specific to the outbreak pathogen and focussed on disease prevention and treatment.

Two of the five studies described measures put in place to prevent infectious disease outbreaks: in one, an outbreak did occur and in the other, no outbreak occurred.^16^^,^^34^

In Denmark, to reduce the occupational risk to people responding to flooding, preventive measures, specifically PPE and hand hygiene, were implemented, but an outbreak of one or more unspecified water-borne diseases did occur.^34^ Following heavy flooding in Bosnia and Herzegovina, Croatia and Serbia, an increased risk of West Nile virus was identified because the floodwater provided a new and expanded habitat for the disease vector—the *Culex pipiens* mosquito—which is common in that region.^16^ The study therefore recommended implementation of an integrated vector management programme including controlling the mosquito larvae and adult mosquitoes, reducing potential breeding sites, vector surveillance and PPE. It also recommended implementing blood safety measures because the virus can be transmitted through transfusion of blood products.^16^ In this case an outbreak did not occur.

The other three studies described measures put in place to respond to an outbreak that had already begun.^24^^,^^25^^,^^29^ When a *S. enterica* outbreak following an earthquake in Italy was traced to contaminated water being used to irrigate crops, the authorities implemented water safety measures to mitigate the outbreak. This included banning the use of contaminated surface water to irrigate crops and implementing specific decontamination processes in local water treatment plants.^24^ In Spain, measures put in place to mitigate the chickenpox outbreak among young children in an emergency camp included isolation of infected individuals, introduction of a vaccine programme among high-risk young people and epidemiological surveillance to monitor the outbreak.^25^ The third study reports that, following an outbreak of *C. hominis* after flooding in Germany, information was provided to the public to prevent the spread of the pathogen.^29^ Initially this involved advising people to boil drinking water before use, although this was reversed when drinking water samples tested negative for the pathogen, and informing them of the risks of swimming in or sunbathing next to rivers where water and land may have been contaminated by heavy rainfall and flooding. Water safety measures, including testing and closure of swimming pools, were also implemented.

### Lessons learned from natural disasters

Lessons learned are described in 11 of the 17 included studies. These lessons relate to prevention—before and during disasters, after disasters and in the longer term (see table 3). Many of the lessons and recommendations were highly specific to the pathogen involved, e.g. recommending the use of prophylactic antibiotics^20^ or the control of the vectors and rodents that spread diseases.^21^^,^^27^

**Table 3:** Lessons learned from natural disasters

		Prevention—before and during disasters					Prevention—after disasters						Long-term prevention	
Document title	Type of natural disaster	Raise healthcare professionals' awareness of post-disaster risks	Advise the public to avoid flood water or bodies of water after flooding/heavy rain	Advise professionals and the public to use PPE around floodwater	Encourage hygiene measures, e.g. hand washing	Vaccination	Prophylactic antibiotics	Surveillance— water sampling	Closure of affected areas	Surveillance— vectors	Vector control	Rodent control	Preparedness plans need flexibility to respond	Improved refuse management
Christova and Tasseva^27^	Flooding		•	•								•		
De Man et al.^28^	Flooding		•	•	•									
Desai et al.^20^	Flooding			•			•							
Gertler et al.^29^	Flooding				•			•	•					
Hubálek et al.^21^	Flooding									•	•			
Kaya et al.^23^	Earthquake					•								
Nigro et al.^24^	Earthquake							•						
Pérez-Martín et al.^25^	Earthquake	•											•	
Radl et al.^22^	Flooding		•											
Socolovschi et al.^32^	Flooding	•												•
Wójcik et al.^34^	Flooding			•	•									

### Prevention before and during disasters

Recommendations included advising professionals and the public to use PPE when coming into contact with floodwater^20^^,^^27^^,^^28^^,^^34^ and advising the public to avoid contact with floodwater and bodies of water after extreme heavy rainfall or flooding.^22^^,^^27^^,^^28^ Specifically, it is suggested that organizers of events such as triathlons should take prior weather conditions into consideration and either cancel events or advise participants about the risks of exposure to water and disease prevention. Other recommendations included encouraging people to implement hygiene measures—such as hand washing—to prevent infection and spread,^28^^,^^29^^,^^34^ raising awareness among health care professionals about the risk of infectious diseases following natural disasters, so that they can identify cases more quickly and advise patients accordingly,^25^^,^^32^ and implementing vaccination programmes to prevent the spread of infectious diseases, especially when people are displaced into temporary accommodation.^23^

### Prevention after disasters

There were also recommendations for measures to prevent infectious diseases after a disaster has occurred. These included conducting vector surveillance, testing water samples where there is a risk of contamination and closing affected areas, e.g. swimming pools or flooded areas, to the public.^21^^,^^24^^,^^29^

### Long-term prevention

Only two studies considered long-term prevention. One highlighted the need for emergency preparedness plans to be flexible enough to incorporate unforeseen circumstances.^25^ In this case, an outbreak of chickenpox at an emergency camp, the plan needed to be flexible enough to incorporate implementation of an unforeseen vaccination programme. The other study made a recommendation that was specific to an outbreak of leptospirosis caused by heavy rains and exacerbated by a strike by refuse collectors that encouraged rat populations.^32^ The recommendation was to improve refuse management.

## Discussion

This review identified 17 studies that met the inclusion criteria. The range of study designs (see table 1) precluded a quantitative meta-analysis and a formal assessment of quality for all studies. In addition, most studies focussed on the cause and impact of infectious disease outbreaks following natural disasters; very few considered prevention, mitigation or control measures.

One limitation of this review is publication bias. While there were studies included in this review that reported on post-disaster settings where no disease outbreaks occurred (table 1), we acknowledge that this study will be biased towards events where an outbreak occurred. This is due to the search strategy used and because publication bias makes it more likely that events with outbreaks would be reported on.

Publication bias also prevented a meta-analysis: given the lack of a comparison group (no disaster) it was not possible to quantitatively summarize the risk of infectious disease events following these disasters. Further research is required to assess the relative frequency of disease outbreaks in post-disaster settings both in Europe and globally, so as to ensure that any preparedness measures implemented are commensurate to the risks involved in specific settings. Activities such as establishing protocols for implementing health registries in disaster settings are important in this regard, for capturing information about the breadth of health outcomes and risk factors in post-disaster settings is essential for the establishment of preparedness and response strategies.^37^

Of the 17 included studies, only 11 reported on the cascading risk pathways of infectious disease outbreaks following earthquakes or floods. These outbreaks were due to food- and water-borne diseases and zoonoses, emerging and vector-borne diseases and vaccine-preventable diseases. Although the absolute number of people affected by these infectious disease outbreaks is low, these outbreaks represent a relative increase in cases compared with usual annual case numbers. As climate change contributes to an increased frequency of flooding, the risk of post-disaster infectious disease outbreaks may rise, as flooding was the most common natural disaster event leading to infectious disease outbreaks identified in this review.

Among food- and water-borne diseases and zoonoses, ‘leptospirosis’ was the most common pathogen described. Although ‘leptospirosis’ is relatively uncommon in Europe, ECDC surveillance suggests that it is increasing in many countries, with some reporting a nearly 3-fold rise in the number of cases in 2014 as compared with the average from the previous 4 years.^38^ Exposure to ‘leptospirosis’ can be exacerbated by heavy rainfall and flooding, and the number of flood events in Europe is increasing and is expected to continue to increase in coming years.^17^

Some population groups may be more vulnerable or at risk of post-disaster infectious diseases than others, depending on the infectious disease concerned, because of factors including vaccination policy, post-disaster living conditions or exposure to environmental risks, e.g. those working in agriculture or those involved in clean-up operations. The latter may include first responders who are not professionals and who are, therefore, less well informed about the potential health risks.

Most of the reported measures taken or recommended to prevent, mitigate and control outbreaks are disease specific. However, our review also identified recommendations about raising awareness among the public and professional awareness of the health risks following natural disasters, to improve recognition of infectious diseases and prevention of the spread of infection. Awareness raising could include avoiding exposure to bodies of water and areas of land that may have been contaminated by sewage due to heavy rainfall and floods, and advising people about how to protect themselves when clearing floodwater from their land or property.

## Conclusions

The cascade effects of natural disasters, such as earthquakes and floods, include outbreaks of infectious disease. The growing frequency and complexity of these events, and the projection that climate change will lead to an increase in extreme weather events (e.g. floods)^39^ while also affecting infectious disease transmission^40^ in Europe in the coming century highlights the importance of improving our understanding of the links and causal pathways between natural disasters and infectious disease outbreaks so as to strengthen preparedness planning and measures to mitigate and control outbreaks. The findings of this review suggest that enhancing data collection and incorporating infectious disease prevention and control measures into natural disaster preparedness and response planning could help to prevent and mitigate outbreaks that can cause death and illness. In addition, the risk of infectious diseases and relevant control measures need to be communicated clearly to the public—particularly to those who may be at elevated risk because of their age, occupation or leisure activities—as well as to health care and other professionals.

## Funding

The research conducted in this article was funded by the European Centre for Disease Prevention and Control and conducted by Bazian, Economist Intelligence Unit, under Specific Contract No. 7, ECD.7947.

*Conflicts of interest*: None declared.

**Key points**

- Elucidating the pathways through which cascading effects lead to negative public health outcomes such as infectious disease outbreaks is essential for informing the design of cross-sectoral preparedness strategies.
- The most reported driver of disease outbreaks was heavy rainfall, which led to cross-connections between water and other environmental systems, leading to the contamination of rivers, lakes, springs and water supplies.
- Post-disaster living conditions, including overcrowding and shared use of bathroom facilities, were connected to two outbreaks following earthquakes.
- Incorporating infectious disease prevention and control measures into natural disaster preparedness and response planning could help to prevent and mitigate outbreaks that can cause death and illness.

## Supplementary Material

ckz111_Supplementary_Data
