# Supplementary material for: Natural disasters and infectious disease in Europe: a literature review to identify cascading risk pathways
Source: Eur J Public Health. 2020 Jun 6;30(5):928–35. doi: 10.1093/eurpub/ckz111 (PMC7536539; doi:10.1093/eurpub/ckz111)
Supplement: ckz111_Supplementary_Data [file ckz111_supplementary_data.docx]

#### **Table S1**. Embase.com search strategy:

| Facet | Search | Search terms | Results |
| --- | --- | --- | --- |
| Europe region | 1 | eu:ti,ab OR ((europe* NEAR/3 (union OR community)):ti,ab) OR austria*:ti,ab OR belgi*:ti,ab OR bulgaria*:ti,ab OR croatia*:ti,ab OR cyprus:ti,ab OR cypriot:ti,ab OR ((czech NEAR/3 republic):ti,ab) OR czech*:ti,ab OR denmark:ti,ab OR danish:ti,ab OR estonia*:ti,ab OR finland:ti,ab OR finnish:ti,ab OR france:ti,ab OR french:ti,ab OR german*:ti,ab OR greece:ti,ab OR greek:ti,ab OR hungary:ti,ab OR hungarian:ti,ab OR ireland:ti,ab OR irish:ti,ab OR ital*:ti,ab OR latvia*:ti,ab OR lithuania*:ti,ab OR luxembourg*:ti,ab OR malta:ti,ab OR malteste:ti,ab OR malti:ti,ab OR netherland*:ti,ab OR poland:ti,ab OR polish:ti,ab OR portug*:ti,ab OR romania*:ti,ab OR slovakia*:ti,ab OR slovenia*:ti,ab OR spain:ti,ab OR spanish:ti,ab OR sweden:ti,ab OR swedish:ti,ab OR britain:ti,ab OR british:ti,ab OR wales:ti,ab OR scotland:ti,ab OR england:ti,ab OR 'united kingdom':ti,ab OR uk:ti,ab OR gb:ti,ab OR iceland*:ti,ab OR liechtenstein*:ti,ab OR norway:ti,ab OR norwegian:ti,ab OR albania*:ti,ab OR bosnia*:ti,ab OR kosov*:ti,ab OR macedonia*:ti,ab OR montenegro*:ti,ab OR serbia*:ti,ab OR turkey:ti,ab OR turkish:ti,ab | 1597308 |
|  | 2 | 'european union':de OR austria:de OR belgium:de OR bulgaria:de OR croatia:de OR cyprus:de OR 'czech republic':de OR denmark:de OR estonia:de OR finland:de OR france:de OR germany:de OR greece:de OR hungary:de OR ireland:de OR italy:de OR latvia:de OR lithuania:de OR luxembourg:de OR malta:de OR netherlands:de OR poland:de OR portugal:de OR romania:de OR slovakia:de OR slovenia:de OR spain:de OR sweden:de OR 'united kingdom'/exp OR 'iceland'/de OR 'liechtenstein'/de OR 'norway'/de OR 'albania'/de OR 'bosnia and herzegovina'/exp OR 'kosovo'/de OR 'macedonia (republic)'/de OR 'montenegro (republic)'/de OR 'serbia'/exp OR 'turkey (republic)'/de | 201284612 |
| Infectious diseases | 3 | ('infectious disease*' NEAR/5 (outbreak* OR epidemic*)):ti,ab | 1856 |
|  | 4 | ('communicable disease*' NEAR/5 (outbreak* OR epidemic*)):ti,ab | 329 |
|  | 5 | 'epidemic'/de OR 'communicable diseases'/de OR 'emerging infectious disease'/de OR 'vector borne disease'/de OR 'zoonosis'/de OR 'food poisoning'/exp OR 'zoonosis'/exp OR 'influenza'/exp OR 'respiratory virus'/de OR 'human immunodeficiency virus'/exp OR 'sexually transmitted disease'/exp OR 'virus hepatitis'/exp OR 'tuberculosis'/exp OR 'vaccine preventable disease'/de OR 'norovirus'/exp OR 'leishmaniasis'/exp OR 'gastroenteritis'/exp OR 'pneumonia'/exp OR 'respiratory tract infection'/exp OR 'leptospirosis'/de OR 'vibriosis'/exp OR 'diarrhea'/exp OR 'escherichia coli'/exp OR 'salmonella'/exp OR 'measles'/exp OR 'meningitis'/exp OR 'malaria'/exp OR 'dengue'/exp OR 'tetanus'/de | 2103304 |
|  | 6 | 'anti?microbial resistan*':ti,ab OR 'health?care?associated infection*':ti,ab OR 'emerg* disease*':ti,ab OR (((vector* NEAR/2 borne):ti,ab) AND disease*:ti,ab) OR 'food?borne disease*':ti,ab OR 'water?borne disease':ti,ab OR zoonos*:ti,ab OR influenza:ti,ab OR 'respiratory virus*':ti,ab OR hiv:ti,ab OR 'sexually transmitted infection*':ti,ab OR 'sti':ti,ab OR 'viral hepatitis':ti,ab OR 'tuberculosis':ti,ab OR 'tb':ti,ab OR 'vaccine?preventable disease?':ti,ab OR norovirus:ti,ab OR leishmaniasis:ti,ab OR gastroenteritis:ti,ab OR pneumonia:ti,ab OR flu:ti,ab OR 'respiratory infect*':ti,ab OR 'hepatitis a':ti,ab OR 'hepatitis e':ti,ab OR leptospirosis:ti,ab OR cholera*:ti,ab OR diarrhoea*:ti,ab OR diarrhea*:ti,ab OR 'e coli':ti,ab OR 'escherichia coli':ti,ab OR 'vibrio':ti,ab OR salmonella:ti,ab OR measles:ti,ab OR meningitis:ti,ab OR malaria:ti,ab OR dengue:ti,ab OR tetanus:ti,ab OR 'clostridium tetani':ti,ab | 1582531 |
|  | 7 | (zoono* NEAR/5 (outbreak* OR epidemic*)):ti,ab | 410 |
|  | 8 | #3 OR #4 OR #5 OR #6 OR #7 | 2553014 |
| Disasters – earthquakes & flooding | 9 | 'earthquake'/de OR earthquake*:ti,ab | 9165 |
|  | 10 | 'flooding'/de OR flood*:ti,ab | 16354 |
|  | 11 | (natural NEAR/3 (disaster* OR catastoph*)):ti,ab | 3717 |
|  | 12 | ((natural NEAR/3 (disaster* OR catastoph*)):ti,ab) OR 'natural disaster'/de OR 'disaster'/de | 24674 |
|  | 13 | #9 OR #10 OR #11 OR #12 | 44785 |
| European countries combined | 14 | #1 OR #2 | 2162444 |
| Infectious diseases + Disasters + Europe | 15 | #8 AND #13 AND #14 | 323 |
| Date + language limitations | 16 | #15 AND (2005:py OR 2006:py OR 2007:py OR 2008:py OR 2009:py OR 2010:py OR 2011:py OR 2012:py OR 2013:py OR 2014:py OR 2015:py OR 2016:py OR 2017:py) AND [english]/lim | 198 |
